# Numerical Investigation of Prefabricated Utility Tunnels Composed of Composite Slabs with Spiral Stirrup-Constrained Connection Based on Damage Mechanics

**DOI:** 10.3390/ma15186320

**Published:** 2022-09-12

**Authors:** Qinghua Wang, Guobin Gong, Jianli Hao, Yuanfeng Bao

**Affiliations:** 1 Department of Structural Engineering, Tongji University, Shanghai 200092, China; 2 School of Architectural Engineering, Nantong Vocational University, Nantong 226007, China; 3 Department of Civil Engineering, Xi’an Jiaotong-Liverpool University, Suzhou 215123, China; 4 Suzhou Integrated Infrastructure Technology Research Institute (SITRI), Suzhou 215000, China

**Keywords:** precast utility tunnel, concrete damage, spiral stirrup, finite element, ductility

## Abstract

This paper investigates prefabricated utility tunnels composed of composite slabs with a spiral stirrup-constrained connection, considering material nonlinearity with concrete damage. An experiment was set up based on the prototype of a practical utility tunnel project, and the results were compared with finite element (FEM) simulation results with reasonable agreement obtained. The parametric analysis was carried out considering variations of seam location, haunch height and reinforcement, and embedment depth, using FEM simulations. It is found that, as with the increase in seam distance above haunch, the load capacity increases slightly, while the ductility does not vary much. The haunch height is not found to have an apparent effect on stiffness, load capacity or ductility. The increase in the embedment depth can enhance both the yield and peak loads while decreasing the ductility. A simplified method is proposed for evaluating the seismic performance in terms of deformation coefficient considering ductility demand, based on three different methods for calculating interaction coefficients considering soil–structure interactions. The findings from this investigation provide theoretical and practical guidance for underground engineering design of prefabricated utility tunnels.

## 1. Introduction

Underground utility tunnels integrate various engineering pipelines, such as electricity, communication, gas, heating, water supply and drainage, which have special inspection and hoisting ports to implement unified planning and management [1,2,3]. These tunnels have become critical infrastructure to ensure the smooth operation of modern cities. In recent years, with the development of China’s economy and process of urbanization, the construction of utility tunnels has developed rapidly throughout the country [4,5].

According to the fabrication materials, utility tunnels can be divided into those built of concrete and those built of steel [6]. Concrete utility tunnels are more popular and widely used, and they are either cast-in-place (CIP) or prefabricated tunnels. The main components of a prefabricated utility tunnel are cast in a factory and assembled onsite to create a whole utility tunnel. The construction period for prefabricated utility tunnels is relatively short, with good and easy quality control and remarkable environmental protection and energy saving [7,8]. Prefabricated concrete utility tunnels, which are the trend in China, are mainly of three types according to the assemblage methods: integrated prefabricated utility tunnels (IPUT), prefabricated utility tunnels composed of composite slabs (PUTCCS), and prefabricated utility tunnels composed of groove-shaped elements (PUTCGE) [9].

The focus of this investigation is PUTCCS, which comprises double-sided composite sidewalls, a composite top slab, and a cast-in situ bottom slab or sometimes composite bottom slab, all of which are then connected to form a monolithic structure by post-casting concrete on site. Researchers have extensively investigated the seismic performance of PUTCCS. Wei et al. [10] took the volume ratio of stirrups and anchorage length of the longitudinal reinforcement as parameters and carried out seismic performance tests of the joints of PUTCCS. They found that adoption of stirrups in the core region of the joint can enhance both the shear and moment capacities of the members. Yang et al. [11] carried out low-cycle repeated loading tests of scaled models of the PUTCCS. The test parameters were the height of the haunch and the depth of the overburden soil layer. The results revealed that bending failure occurred in all the specimens, with the limit drift ratio between 1/27 and 1/20, and with relatively good ductility. Wang [12] subjected two full-scale PUTCCS specimens to cyclic loadings, one with an exterior precast top joint and the other with a CIP top joint. It was found that the bearing capacity of the precast specimen was 9.5% lower than that of the CIP specimen.

**Figure 1:**
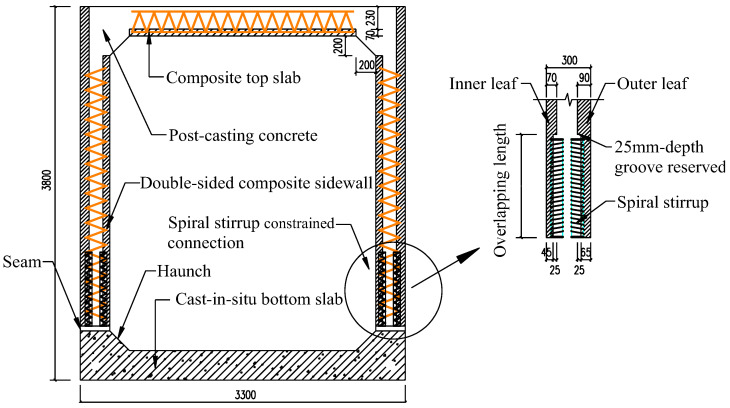
Schematic diagram of composite precast utility tunnel with spiral stirrup-constrained connection.

It should be mentioned that the top and bottom slabs of PUTCCS in the aforementioned research are composite slabs; in actual engineering practice, the CIP bottom slab is more convenient in terms of construction. The connection between the sidewall and the CIP bottom slab of PUTCCS is generally formed by reserving a certain anchorage length with overlapping of the reinforcements between the bottom slab and sidewall. However, this type of connection for PUTCCS is different from the traditional overlapping connection for steel, because, for the former type, the vertical steel bars in the prefabricated sidewalls are not in contact with the steel bars extended upward from the CIP bottom slab. It is therefore difficult to form a valid connection. It is for this purpose that spiral stirrups are used to restrain the overlapping connection, as shown in Figure 1. During prefabrication of the sidewalls, distributed spiral stirrups are imbedded along the vertical steel bars on the inner and outer leaves of the sidewalls, and a 25 mm-depth groove is reserved so that the steel bars extended upward from the bottom slab can be put in place. In this way, the vertical reinforcements for the sidewall and the extended reinforcements from the bottom slab are all inside the spiral stirrups, forming an effective overlapping connection, and so the overlapping length can be shortened to some degree, due to the restraint effect of the stirrup, as demonstrated in [13,14]. Imai [13] showed that the pull-out capacity of the overlapping connection can be improved by increasing the thickness of the cover and the volume ratio of the stirrups. Jiang et al. [14] conducted overlapping tests of 108 specimens, considering the influences of factors such as steel bar diameter, concrete strength, and overlapping length, and established relationships among these factors for the failure mode of the overlapping connection. They found that the overlapping length can be shortened to one standard anchorage length, subject to such factors as concrete strength, steel strength, steel bar diameter, and type of steel; see also DB23/T1813-2016 [15].

Little research can be found on the seismic performance of the spiral stirrup-constrained connection for PUTCCS, except Tian [16], who performed a few joint static performance tests and found that a constrained spiral stirrup improved the connection performance. However, there have been no reported studies on the seismic performance of the overall structure of PUTCCS with a spiral stirrup-constrained connection, especially when soil–structure interactions are involved.

In addition, there are not many design specifications for utility tunnels. Many structural design codes or guidelines [17,18,19,20] do not include specifications of design or construction requirements of precast concrete utility tunnels. Some detailed regulations and descriptions of the static and seismic design of the utility tunnel are provided in [21], but mainly for the CIP concrete utility tunnel and do not include that of the precast utility tunnel. GB50838-2015 [22] proposes a design method only for precast utility tunnels composed of groove-shaped elements. It can be concluded that there is a lack of studies on the structural design of precast utility tunnels composed of composite slabs.

This investigation takes a practical underground utility tunnel project, located on Hongtu Street in Harbin, China, as the prototype. The ABAQUS software is used to establish a nonlinear finite element model of PUTCCS with a spiral stirrup-constrained connection. Parametric analysis is performed with a focus on the influences of seam location, haunch height and reinforcement and embedment depth on the overall structural behavior of PUTCCS, with exploration of the seismic performance in terms of ductility demand considering soil–structure interactions.

## 2. Overview of the Experiment

**Figure 2:**
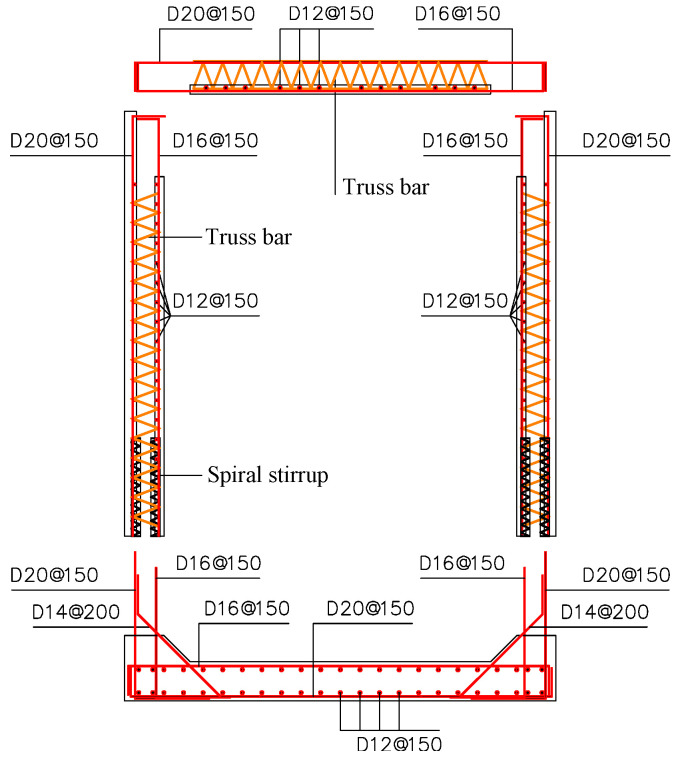
Reinforcement details of specimen.

The overall dimension of the concrete specimen (C40) is 3800 mm high, 3300 mm wide, and 800 mm thick. The reinforcement layout of the test specimen is shown in Figure 2. The reinforcement mainly comprises longitudinal bars, truss bars and spiral stirrups. HRB400 steel is used for the longitudinal bars and HPB300 steel is used for the spiral stirrups. The outer sides for the vertical walls and top and bottom slabs have a cover thickness of 50 mm, with a diameter of 20 mm for the longitudinal bars. The inner sides for the vertical walls and top and bottom slabs have a cover thickness of 30 mm, with a diameter of 16 mm for the longitudinal bars. A truss bar system comprises one straight bar (diameter of 10 mm) at the top, two straight bars (diameter of 10 mm) at the bottom, and a V-shaped tie bar (diameter of 6 mm) connecting the straight bars. For the truss bar system, HRB400 steel is used for the straight bars and HPB300 steel is used for V-shaped tie bars. Two truss bar systems with a spacing of 400 mm are arranged longitudinally in each sidewall, as well as in the top slab. There are two types of spiral stirrups. One type is with a diameter of 6 mm, a spacing of 50 mm, a height of 600 mm, and an overall section diameter of 70 mm, which is for the overlapping of the outer longitudinal bars. The second type is with a diameter of 4 mm, a spacing of 40 mm, a height of 480 mm, and an overall section diameter of 70 mm, which is for the overlapping of the inner longitudinal bars. The mechanical properties of concrete and steel reinforcements, were tested in the structural laboratory of Tongji University according to the standard test method specified in Chinese code GB/T 50081-2019 [23] and GB/T 2281-2010 [24].

**Figure 3:**
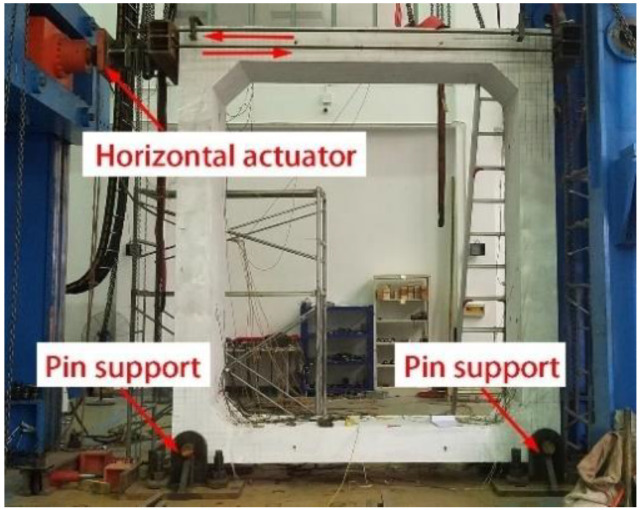
Test setup.

**Figure 4:**
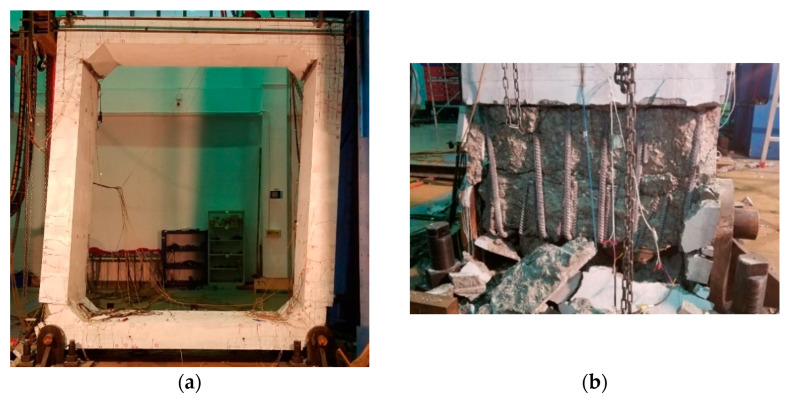
Failure pattern of specimen. (**a**) The whole structure; (**b**) Bottom joint.

Cyclic horizontal loadings were applied at the top slab via a hybrid-controlled loading mode as prescribed by the Chinese Specification for Seismic Test of Buildings (JGJ/T101-2015) [25]. The pin supports are set on both the left and right edges of the bottom slab. Some details about the loading can be found in [26]. The loading setup is shown in Figure 3, with an attempt to model PUTCCS imbedded in the ground under the action of an earthquake [27,28]. Figure 4 shows the failure mode of the specimen, which indicates a bending failure as characterized by crushing of concrete observed mainly at the bottom joint connecting the outer sides of the side wall and bottom slab, with the bending bars exposed. The general procedure for experimental data processing and analysis can be found in [29,30].

## 3. Finite Element Modeling and Verification

### 3.1. Finite Element Modeling

**Figure 5:**
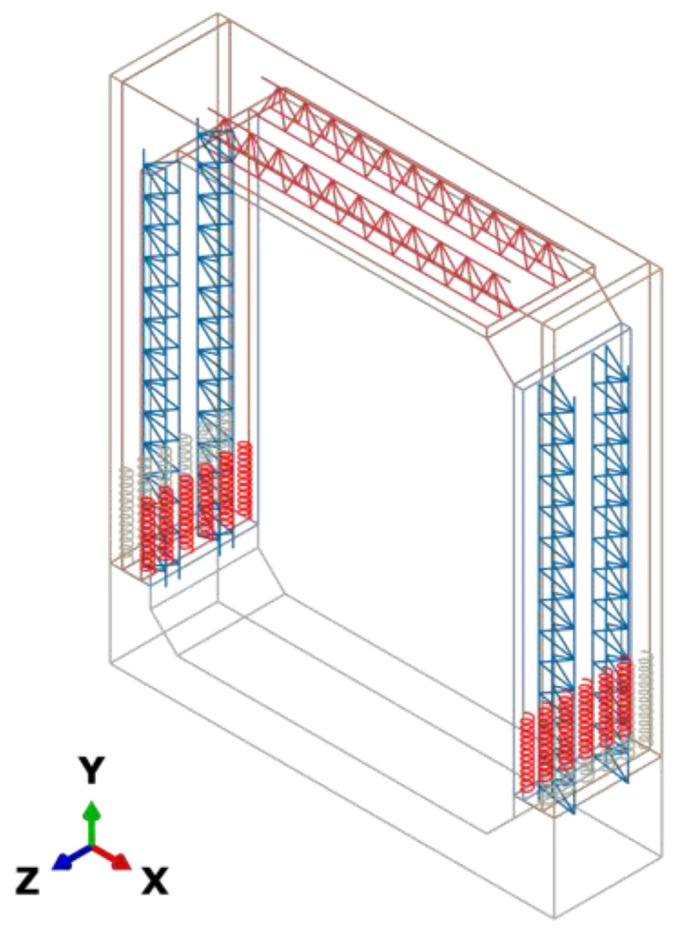
Finite element model.

Finite element method is a powerful numerical method for solving differential equations in an approximate manner [31,32]. Based on the above testing prototype, a finite element model of PUTCCS with a spiral stirrup-constrained connection using ABAQUS is established, as shown in Figure 5.

**Figure 6:**
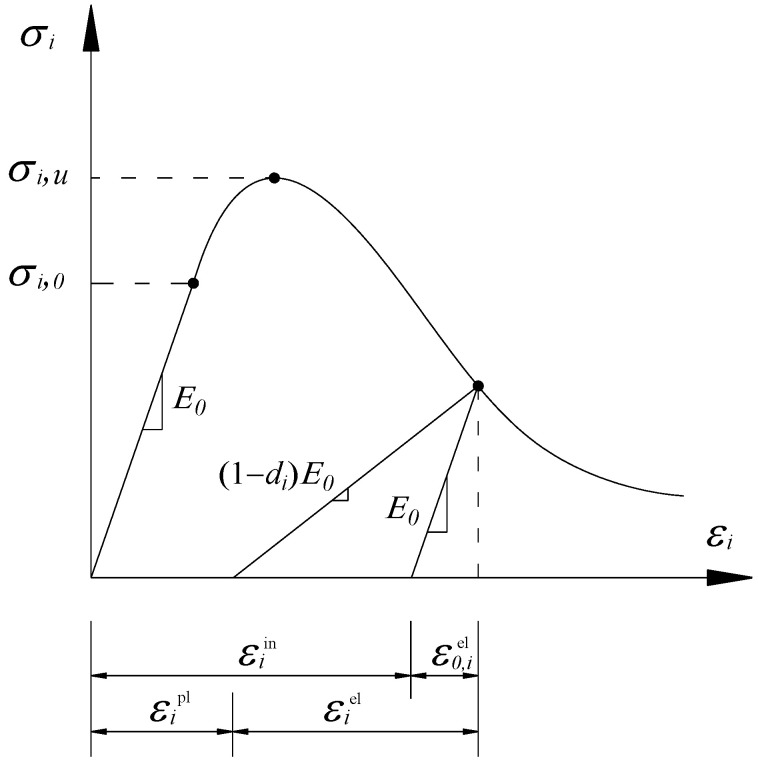
Constitutive relation of concrete.

The damage-plastic material model is adopted to simulate the constitutive stress–strain relationship of concrete [33,34]. The bilinear elastic-plastic material model is adopted to simulate the constitutive relationship of the reinforcements. The constitutive relationship curve of concrete under uniaxial compression is shown in Figure 6, where $\varepsilon_{,i}^{el}$ corresponds to the elastic strain without damage during unloading, $\varepsilon_{i}^{el}$ is the elastic strain with damage during unloading, $\varepsilon_{i}^{pl}$ is the plastic strain after unloading, and $\varepsilon_{i}^{in}$ is the inelastic strain (part of inputs in Abaqus). In the figure, *E*_0_ indicates the initial elastic modulus with a value of 32,500 MPa, *d_i_* indicates plastic-damage factor ranging from 0 to 1, wherein “0” indicates no damage, while “1” indicates full damage (strength completely lost). It should be mentioned that the inelastic strain $\varepsilon_{i}^{in}$ is not equal to plastic strain $\varepsilon_{i}^{pl}$ when *d_i_* is not zero. In this discussion concerning Figure 6, uniaxial tension could also be imposed, with a similar but different pattern, details of which can be found in [33].

The Abaqus software automatically converts the inelastic strain values to the plastic strain values using the following Equation (1) [33]:

(1) $$\varepsilon_{i}^{pl}=\varepsilon_{i}^{in}-(\varepsilon_{i}^{el}-\varepsilon_{0,i}^{el})=\varepsilon_{i}^{in}-\frac{d_{i}\sigma_{i}}{(1-d_{i})E_{0}}$$

where the subscript “*i*” refers to compression or tension.

According to Sidoroff’s principle of energy equivalence, the elastic residual energy $W_{d}^{e}$ produced by the stress acting on the damaged material is assumed to be the same in form as the elastic residual energy $W_{0}^{e}$ acting on an imagined non-damaged material.

For the imagined non-damaged material:

(2) $$W_{0,i}^{e}=\frac{\sigma_{i}{}^{2}}{2E_{0}}$$

For the damaged material which has the same form as Equation (2):

(3) $$W_{d,i}^{e}=\frac{{\bar{\sigma }}_{i}{}^{2}}{2E_{d}}$$

where ${\bar{\sigma }}_{i}$ is the “effective” tensile and compressive cohesion stress, and defined as in Equation (4), where *E_d_* is the secant modulus:

(4) $${\bar{\sigma }}_{i}=\frac{\sigma_{i}}{1-d_{i}}$$

From Equations (2)–(4), we obtain:

(5) $$E_{d}=E_{0}{\left(1-d_{i}\right)}^{2}$$

Substituting

(6) $$\sigma_{i}=E_{d}\varepsilon_{i}$$

into Equation (5) leads to:

(7) $$d_{i}=1-\sqrt{\frac{\sigma_{i}}{E_{0}\varepsilon_{i}}}$$

Table 1 shows the input parameters for the concrete damage model used in the simulations, based on the concrete’s stress–strain relationship as specified for C40 in the Chinese concrete code GB50010 [35].

The linear truss element type of T3D2 is used to model the steel bars (reinforcements) and the solid element type of C3D8R is used to model the concrete. The automatic meshing is used with an element division of 50 mm. The numbers of truss and solid elements are 9172 and 26,208, respectively. Automatic time step in Abaqus is adopted, which is sufficient for the simulations reported in this work since only material nonlinearity is involved. Good convergency of the results is obtained. The reinforcement elements (T3D2) are coupled to the concrete elements (C3D8R) through “embedded” constraints, ignoring the bond-slip between reinforcement and concrete.

The experimental results indicate obvious cracking on the seam interface between the bottom of the side wall and the top of the bottom slab, while no obvious cracking or slip was observed in the double-sided sidewalls and the composite top slab combined with the post-casting layers. Therefore, contact surface interaction with “surface-to-surface contact” is adopted for the horizontal seam surface at the bottom of the sidewall. In terms of the contact properties, “hard contact” is adopted along the normal contact direction with separation allowed after contact, and “friction contact” is adopted along the tangential contact direction with a friction coefficient of 0.6 between the rough concrete surfaces.

### 3.2. Model Validation

In the simulations performed in this investigation, monotonic loading with displacement control is applied to the utility tunnel, which is different from the cyclic loading in the experiments as described in Section 2. However, it is well known that hysteretic loop curves can be obtained from the cyclic loading with a well-defined skeleton curve (response envelope) connecting the peak points of each loading in the same direction on the hysteretic loop curve. Such a skeleton curve can clearly reflect the strength and deformation of the structure. It is on this basis that monotonic loading is adopted in the simulations.

**Figure 7:**
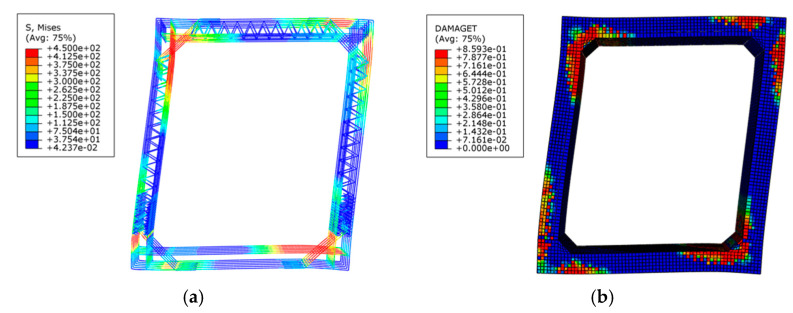
Failure pattern of specimen in FEM. (**a**) Reinforcement stress (MPa); (**b**) Concrete damage factor.

Figure 7a shows the contour of the Mises stress invariant (equal to the axial stress under uniaxial loading) for the reinforcement, where the inner and outer longitudinal reinforcement and the haunch reinforcement at the corner of the utility tunnel all yield, which conforms to the experimental observations. Figure 7b shows the contour of damage factors (describing the damage degree) for the concrete, where it can be seen the main damage occurs in the corners of the utility tunnel with a damage factor up to 0.859, which is also consistent with experimental observations.

**Figure 8:**
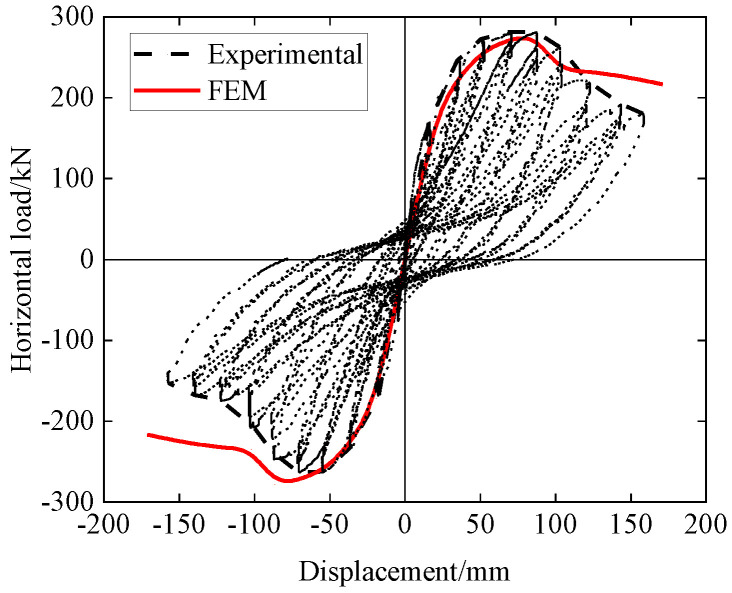
Comparison of load–displacement curves for PUTCCS between FEM and experimental results.

Figure 8 shows comparison of load–displacement curves for PUTCCS between FEM and experimental results, wherein the curve for the experimental results is a skeleton curve of the hysteretic loop curves. The positive and negative peak loads, and ductility from the FEM and experimental values are listed and compared in Table 2, where it can be seen that good agreement is obtained between the FEM and experimental results in terms of the peak load values and ductility.

**Figure 9:**
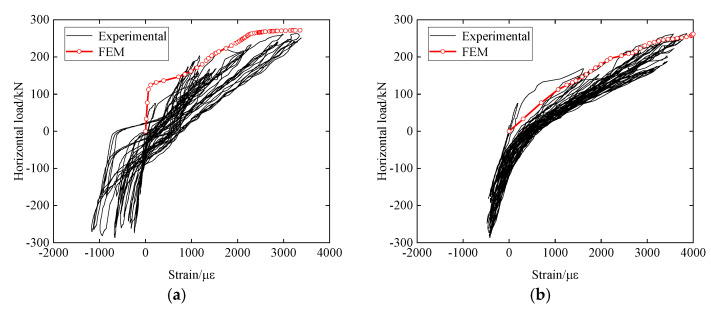
Comparison of load-strain curves for reinforcement between FEM and experimental results. (**a**) Outer reinforcement at the upper end of sidewall; (**b**) Outer Reinforcement at the lower end of sidewall.

Figure 9a,b show comparisons of load-strain curves between FEM and experimental results, for the outer reinforcements at the upper and lower ends of the sidewall, respectively, where it can be seen that the load-strain curves from FEM can well represent the skeleton curves (response envelopes) from the experiments, which is another way of showing the agreement between the FEM and experimental results.

## 4. Parametric Analysis

**Figure 10:**
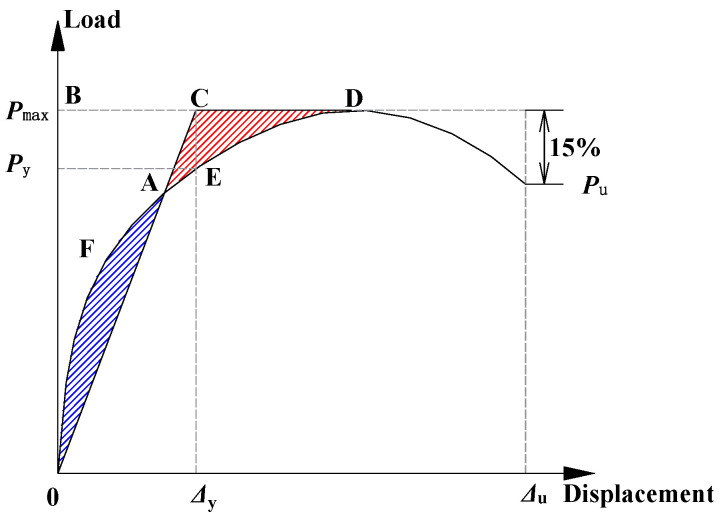
Method used to define yield and ultimate displacements.

Parametric analysis is performed using Abaqus considering the variations of the seam location, haunch height and reinforcement, and embedment depth. The load–displacement curve of the specimen is obtained by FEM simulations, and the structural performance indicators include yield load (*P*_y_), peak load (*P*_max_), yield displacement (Δ_y_), ultimate displacement (Δ_u_), and ductility (*μ*) of the specimen. The yield load and yield displacement are calculated based on the energy method proposed by Park [36]. The basic principle of this energy method is that the curved area A0F is equal to the curved area ACD in Figure 10 and the points C and E can be determined accordingly, the details of which can be found in [36]. The ultimate displacement is taken as the displacement corresponding to 85% of the peak load during the softening stage, and the ductility, i.e., the ductility ratio, is taken as the ratio of the ultimate displacement to the yield displacement.

### 4.1. Seam Location

In order to study the effects of seam locations, four sets of seam locations are used for this investigation, which vary according to the vertical distance between the top of the haunch (for the bottom slab) and the seam. Such vertical distances are 0, 0.5, 1.0, and 1.5 times the wall thickness (300 mm), respectively, as shown in Table 3.

**Figure 11:**
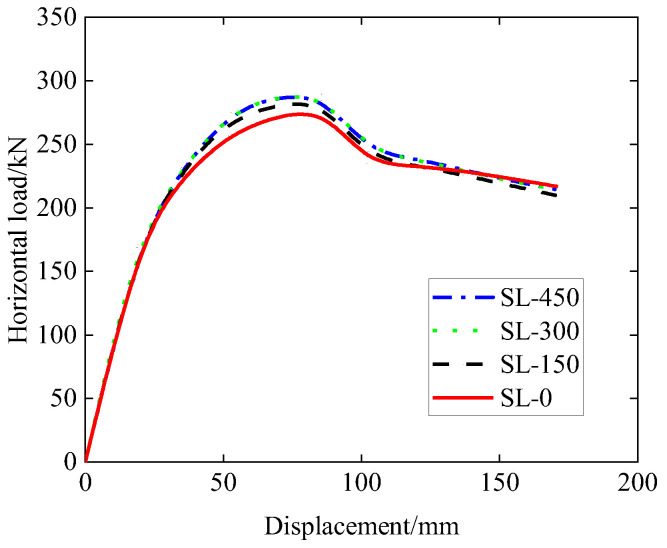
Load–displacement curve (effect of seam location).

The simulation results in terms of the horizontal load–displacement relationship are plotted in Figure 11, and the results in terms of values of the yield load, peak load, yield displacement, ultimate displacement, and ductility are shown in Table 3. It should be noted that the scenario for the SL-0 sample in Figure 11 corresponds to the actual experimental situation with loading in one direction, and the FEM result for SL-0 is also plotted in Figure 8. It can be seen from Figure 11 that all the four samples (with different distances above haunch) have similar load–displacement patterns. All the four samples share the same initial slope of the load–displacement curve before the yield load is reached. As with the increase in the distance above haunch, both the yield and peak loads are found to increase slightly. It can be seen from Table 3 that when the distance above haunch is 0 mm, i.e., when the top of the haunch and the seam coincide, the ductility of the system is the largest, while the ductility does not vary much when the distance above the haunch is from 150 mm to 450 mm.

### 4.2. Haunch Height and Reinforcement

In order to study the effects of haunch height and reinforcement, five sets of haunch height and reinforcement are used for this investigation, which vary according to the haunch height and/or haunch reinforcement (four corners for both the top and bottom slabs). The setting of the haunch height and reinforcement is based on common practice in engineering projects. The haunch height ranges from 0 (indicating no haunch) to 150 mm to 200 mm. The haunch reinforcement ranges from D12@200 to D14@200 to D16@200, where D12@200 refers to reinforcements (HRB400 steel) with a diameter of 12 mm distributed at 200 mm center-to-center distances along the longitudinal direction of the utility tunnel.

**Figure 12:**
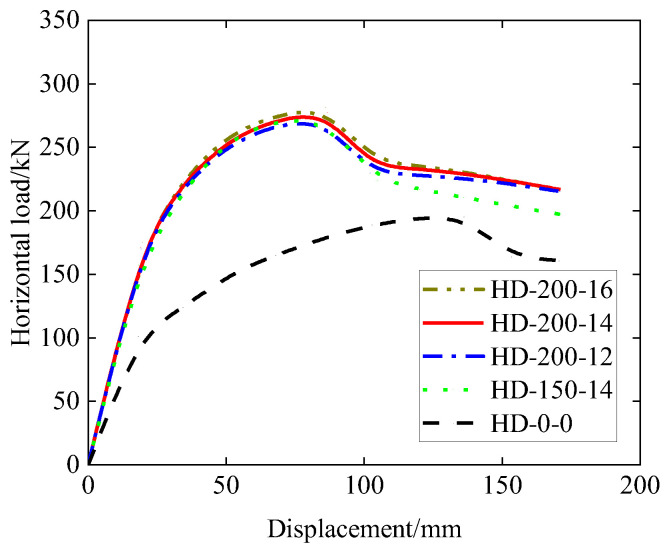
Load–displacement curve (effect of haunch size and reinforcement).

The simulation results in terms of the horizontal load–displacement relationship are plotted in Figure 12, and the results in terms of values of the yield load, peak load, yield displacement, ultimate displacement, and ductility are shown in Table 4. It should be noted that the scenario for the HD-200-14 sample in Figure 12 corresponds to the actual experimental reinforcement situation, and the FEM result for HD-200-14 is also plotted in Figure 8. It can be seen from Figure 12 that the four samples with haunches have similar load–displacement patterns, while the sample without haunches (HD-0-0) has an obviously lower initial stiffness, obviously lower yield and peak loads, and lower ductility (refer to Table 4). Comparing the results of the sample HD-200-14, the peak load capacity for HD-0-0 is found to decrease by 29.6%, and the ductility for HD-0-0 is found to decrease by 16.7%. A comparison of the results of the samples HD-150-14 and HD-200-14 indicates that the increase in the haunch height (from 150 mm to 200 mm) can slightly enhance the load capacity in terms of both yield and peak loads as well as the ductility. A comparison of the results of the samples HD-200-12, HD-200-14 and HD-200-16 indicates that the increase in the reinforcement size from 12 mm to 14 mm to 16 mm (without changing other aspects) can slightly enhance both the yield and peak loads, but slightly decrease the ductility.

### 4.3. Embedment Depth

The embedment depth for a prefabricated utility tunnel in practice is usually not more than 10 m, corresponding to shallow embedment depth, especially when the open-cut method is used for the construction of such tunnels. The change in the embedment depth only involves the change in earth pressure on the top slab of the utility tunnel in the current investigation. The effect of the embedment depth is thus reflected by the loading on the top slab. In order to study the effects of such embedment depths, four sets of embedment depths are considered, which range from 0 m to 2 m to 5 m to 10 m, as shown in Table 5.

**Figure 13:**
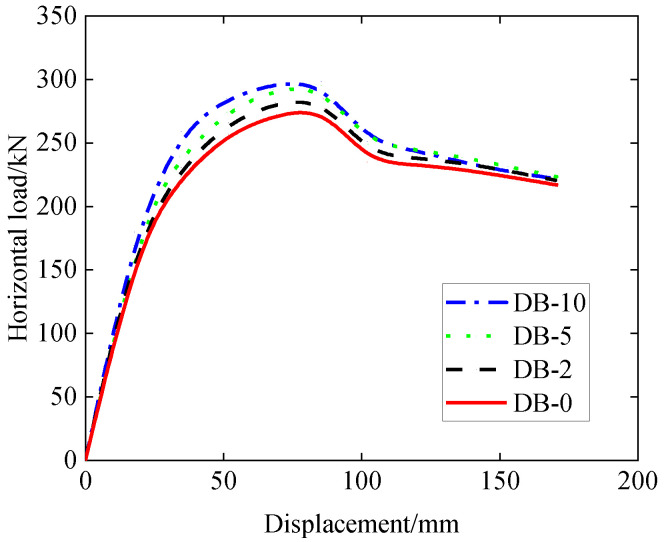
Load–displacement curve (effect of embedment depth).

The simulation results in terms of the horizontal load–displacement relationship are plotted in Figure 13, and the results in terms of values of the yield load, peak load, yield displacement, ultimate displacement, and ductility are shown in Table 5. It should be noted that the scenario for the DB-0 sample in Figure 13 corresponds to the actual experimental situation with loading in one direction, and the FEM result for DB-0 is also plotted in Figure 8. It can be seen from Figure 13 that all four samples have similar load–displacement patterns, and that they share the same initial stiffness in the load–displacement curve before the yield load is reached. As with the increase in the embedment depth, both the yield and peak loads are found to increase gradually, as can be seen in Table 5, while the ductility is found to decrease gradually. Comparison of the results of the samples DB-0 and DB-10 indicates that the increase in the embedment depth from 0 m to 10 m can enhance the load capacity in terms of the peak loads by 7.7% and decrease the ductility by 14.9%. Physically, the increase in the embedment depth in this investigation is equivalent to the increase in the axial/vertical loading of the sidewall, which is like the behavior of a column with combined axial compression and bending, leading to an increase in the horizontal load capacity and decrease in ductility.

## 5. Seismic Performance Evaluation Considering Soil–Structure Interaction

The utility tunnel is a large-scale underground structure, and the soil–structure interaction effect should not be ignored under earthquake actions. The flexibility coefficient method, also known as the soil–structure interaction coefficient method, is a commonly used simplified design method for underground structures under earthquake conditions [37,38,39]. The main core of the calculation steps of this method is:

(8) $$\Delta_{s}=R\Delta_{free-field}$$

where Δ_s_ is the racking deformation of the structure between the top and bottom slabs under earthquake action considering soil–structure interactions, Δ_free-field_ is the free-field deformation under earthquake action, and *R* is the interaction coefficient or racking coefficient. The free-field deformation refers to the deformation of the soil with no structure and no opening in the ground.

The free-field deformation under an earthquake can be calculated according to GB50909-2014 [40]:

(9) $$\Delta_{free-field}=U(z_{1})-U(z_{2})$$

where *U*(*z*_1_) and *U*(*z*_2_) are the horizontal displacements of the soil stratum corresponding to the locations of the top and bottom slabs of the structure under free-field deformation, respectively.

The horizontal displacement of the soil stratum under free-field deformation *U*(*z*) can be obtained from the following:

(10) $$U(z)=\frac{1}{2}U_{max}\cos\frac{\pi z}{2H}$$

where *z* is the depth of the soil stratum, *U*_max_ is the maximum horizontal displacement of the site surface, which can be found with reference to GB50909-2014 [40], and *H* is the vertical distance from the ground surface to the seismic action datum.

There are several methods to calculate the interaction coefficient *R* in the literature, which are all related to the relative stiffness ratio of the soil and the structure and/or Poisson’s ratio of the soil. Wang [41], Penzien [42], and Nishioka [43] proposed Equations (11)–(13), respectively, for calculating *R*, as follows:

(11) $$R=\frac{8K_{r}(1-\nu )}{2K_{r}+(5-6\nu )}\;(\mathrm{Wang}’s\;\mathrm{method})$$

(12) $$R=\frac{4K_{r}(1-\nu )}{K_{r}+(3-4\nu )}\;(\mathrm{Penzien}’s\;\mathrm{method})$$

(13) $$R=\frac{2K_{r}}{K_{r}+1}\;(\mathrm{Nishioka}’s\;\mathrm{method})$$

where ν is the Poisson’s ratio of the soil, and *K*_r_ is the ratio of the soil stiffness (*k*_s_) to the structural stiffness (*k*_st_) for the utility tunnel. Setting a value of 0.5 for ν in Equations (11) and (12) leads to Equation (13). In other words, Nishioka’s method is a special case of either Wang’s method or Penzien’s method by setting ν equal to 0.5.

The soil stiffness is calculated as follows:

(14) $$k_{s}=\frac{LG}{B}$$

where *G* is the shear modulus of the soil, *L* is the width of the cross section of the utility tunnel, and *B* is the height of the cross section of the utility tunnel.

**Figure 14:**
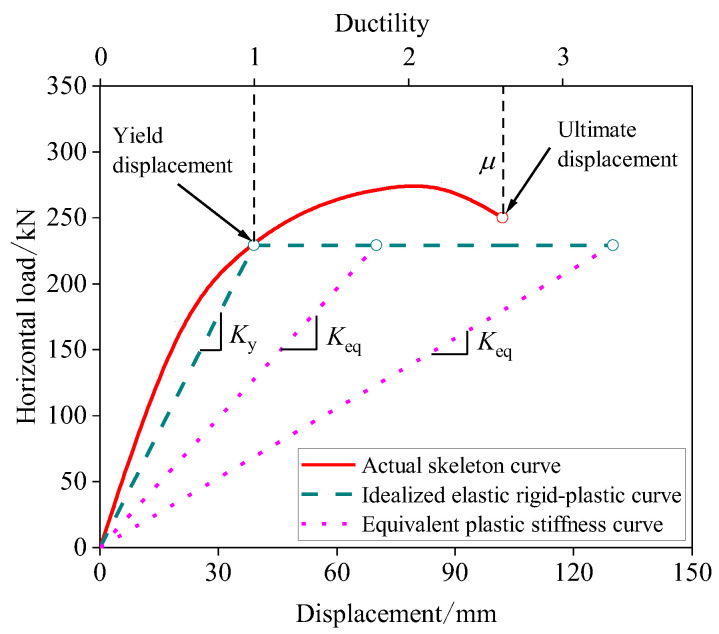
Idealized elastic rigid-plastic curve.

The structural stiffness *k*_st_ can be obtained based on FEM. Pinned supports are set at the lower ends of the sidewall of the structure, and the load–displacement curve can be obtained by applying a concentrated lateral force to the top of the structure and the elastic stiffness is taken as the structural stiffness, see also Wang [41]. However, the method provided by Wang [41] has a limitation in that the structure needs to be elastic. To extend the applicability of the above methodology to the structure with plastic deformation, a simplified method is proposed for the utility tunnel in this investigation, where the load–displacement curve is idealized as an elastic rigid-plastic bilinear line, as shown in Figure 14. In this simplified method, the yield load and yield displacement are determined by using the energy method proposed by Park [36] in relation to the discussion of point A in Figure 10. In Figure 14, *K*_y_ corresponds to the stiffness in the elastic phase in the idealized elastic rigid-plastic model, *K*_eq_ is the secant stiffness corresponding to the plastic phase.

The calculation procedure of the racking deformation can be outlined as follows:

Determine the basic parameters such as site characteristic, dimensions of the utility tunnel, and seismic conditions.

1. Input the initial value *D*_ini_ (can be assumed to be a small trial value for the first attempt) of the racking deformation of the utility tunnel.
2. Calculate the structural stiffness *k*_st_ using the simplified elastic rigid-plastic model.
3. Calculate the soil stiffness *k*_s_ based on Equation (14).
4. Calculate the interaction coefficient *R* by using one of Equations (11)–(13).
5. Calculate the racking deformation Δ_s_ of the structure under earthquake action using Equation (8).
6. If the difference between Δ_s_ in Step 5 and *D*_ini_ in Step 2 is relatively large, meeting
  |*D*−Δ_s_|/*D* ≥ 0.001, increase the initial displacement *D*_ini_ and repeat steps 3 to 6 iteratively until the updated values of Δ_s_ and *D*_ini_ meet |*D*−Δ_s_|/*D* < 0.001.
7. The racking deformation Δ_s_ can then be obtained from the final value of Δ_s_ in Step 6.

**Figure 15:**
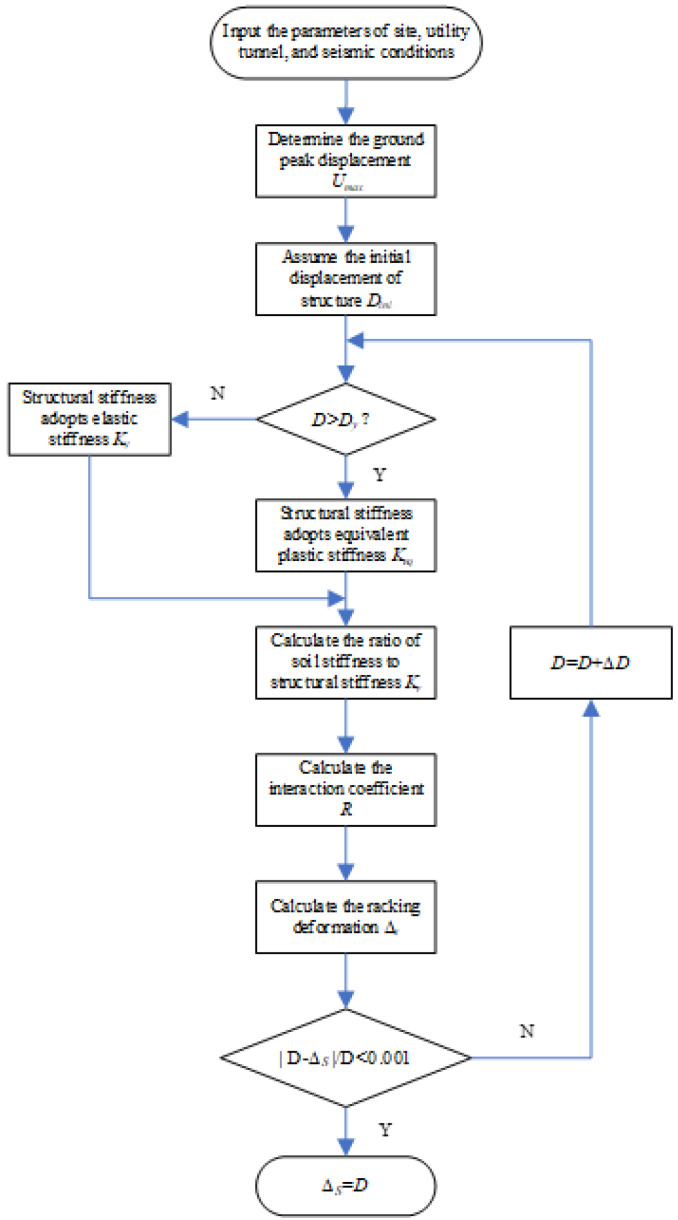
Flow chart of the procedure to obtain racking deformation.

The above procedure can be illustrated as a flow chart shown in Figure 15.

The site characteristic, dimensions of the utility tunnel, and seismic conditions are listed in Table 6. It should be noted that according to seismic precautionary intensity of 8 (0.3 g), the maximum displacement of the site surface is set to 490 mm for analysis [40].

**Figure 16:**
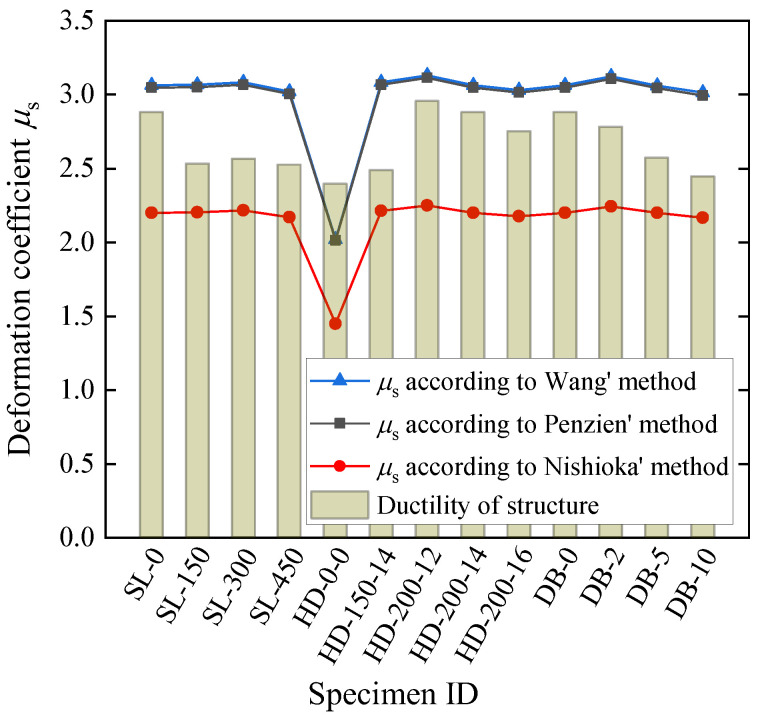
Seismic performance evaluation considering soil–structure interaction.

For the convenience of discussion, we define a deformation coefficient *μ*_s_ as the ratio of the racking deformation Δ_s_ to the yield displacement Δ_y_. Three methods for calculating *R* are used to obtain the final Δ_s_, respectively, following the calculation procedure in Figure 15, and the results are compared considering effects of seam location, haunch height and reinforcement, and embedment depth. The results for the seismic performance evaluation (in terms of the deformation coefficient) considering soil–structure interaction are shown in Figure 16. A summary of the discussions is listed below.

1. The results of the seismic performance in terms of deformation coefficient indicate that Penzien’s and Wang’s methods (with a traditional value of 0.3 for Poisson’s ratio) are almost identical, giving a larger value of *μ*_s_ than that using Nishioka’s method for each sample. This is mainly because Nishioka’s method does not consider the influence of the changes in Poisson’s ratio, which is equivalent to the case where a fixed value of Poisson’s ratio of 0.5 is used in Penzien’s and Wang’s methods. It can be easily verified that from Equations (11) and (12), the interaction coefficient *R* decreases with the increase in Poisson’s ratio. Table 7 shows the interaction coefficient *R* using Wang’s, Penzien’s and Nishioka’s methods, which confirms the above discussion in relation to the effects of change in Poisson’s ratio, since *μ*_s_ is in positive proportion to *R.*
2. The smallest deformation coefficient occurs for the haunch-free specimen HD-0-0 for each of the three methods (Penzien’s, Wang’s, and Nishioka’s), respectively. This is mainly due to the large yield displacement (63.96 mm) of this specimen, which is significantly larger than that of the other specimens ranging from 40.75 mm to 42.12 mm, as shown in Table 3, Table 4 and Table 5. It should be mentioned that specimen HD-0-0′s interaction coefficient *R* is the largest for each of the methods, respectively, due to the small structural stiffness. However, the variation of *R* across the whole specimen list is not much, as can be seen in Table 7. Therefore, the dominant factor affecting the deformation coefficient is the yield displacement for the cases under consideration.
3. If the ductility obtained from the load–displacement curve without considering soil–structure interactions is treated as a limit of the deformation coefficient and applied to the seismic conditions (where soil–structure interactions are normally considered), we can compare the value of deformation coefficient under different scenarios. It can be seen from Figure 16 that the deformation coefficient of each specimen based on Nishioka’s method falls below that corresponding to the structural ductility histogram, while the deformation coefficient for each specimen using Penzien’s and Wang’s methods, lies above that corresponding to the structural ductility histogram, except the very special specimen HD-0-0. In terms of the limit value of the deformation coefficient (ductility), the comparison of the results for the specimen HD-0-0 indicates that a smaller loading capacity and lower ductility without considering soil–structure interactions may correspond to a lower value of deformation coefficient under seismic conditions with soil–structure interactions, and may therefore exhibit a better seismic performance in terms of ductility demand.
4. It should also be mentioned that different methods for calculating *R* may lead to different conclusions in terms of an evaluation of the seismic performance, and further investigations should be made as regards which method provides realistic results.

## 6. Conclusions

This investigation focused on numerical investigations of the seismic performance of prefabricated utility tunnels composed of composite slabs (PUTCCS) with a spiral stirrup-constrained connection. An experiment was set up based on the prototype of a practical utility tunnel, the results of which were compared with FEM simulation (considering material nonlinearity with concrete damage) results with reasonable agreement obtained. The parametric analysis without considering soil–structure interactions was conducted in terms of seam location, haunch height and reinforcement, and embedment depth, based on FEM simulations. A simplified method was proposed for evaluating the seismic performance in terms of deformation coefficient considering ductility demand, based on three different approaches of obtaining interaction coefficient (racking coefficient) considering soil–structure interactions. The main conclusions are as follows.

1. All the samples with varying seam locations share the same initial slope of the load–displacement curve before the yield load is reached. As with the increase in seam distance above haunch, a slight increase in both the yield and peak loads is observed, while the ductility does not vary much.
2. The haunch-free sample has an obviously lower initial stiffness and lower yield and peak loads, and lower ductility in comparison with those for the samples with haunches. The increase in the reinforcement size can enhance both the yield and peak loads, but decrease the ductility.
3. The increase in the embedment depth can enhance both the yield and peak loads while decreasing the ductility.
4. The seismic performance in terms of deformation coefficient considering soil–structure interactions indicates that Penzien’s and Wang’s methods are almost identical, giving a larger deformation coefficient than that using Nishioka’s method.
5. The smallest deformation coefficient occurs for the haunch-free specimen for each of the three methods (Penzien’s, Wang’s, and Nishioka’s), respectively. The dominant factor affecting the deformation coefficient is the yield displacement.

## Figures and Tables

**Table 1:** Input parameters for the concrete damage model in Abaqus.

*x*	0.4	0.6	0.8	1.0	1.2	1.4	1.6	1.8	2.0	2.2	2.4	2.6	2.8	3.0	5.0	8.0	10.0
$\varepsilon_{c}^{el}$/με	0	232	469	766	1115	1499	1892	2280	2661	3032	3395	3752	4103	4449	7778	12,622	15,826
*σ_c_*/MPa	18.16	23.47	26.10	26.80	25.79	23.64	21.22	18.93	16.91	15.18	13.71	12.45	11.39	10.47	5.65	3.28	2.56
*d_c_*	0	0.130	0.206	0.280	0.355	0.428	0.494	0.549	0.596	0.635	0.668	0.696	0.720	0.740	0.852	0.911	0.930
$\varepsilon_{t}^{el}$/με	—	—	—	0	58	88	116	143	169	194	218	241	264	287	505	822	1032
*σ_t_*/ MPa	—	—	—	2.39	2.18	1.88	1.63	1.42	1.26	1.14	1.03	0.95	0.88	0.82	0.50	0.34	0.28
*d_t_*	—	—	—	0	0.267	0.369	0.451	0.516	0.568	0.609	0.643	0.672	0.696	0.716	0.828	0.888	0.909

Note: *x* represents the ratio of the compressive or tensile strain of concrete to the strain corresponding to the peak stress.

**Table 2:** Comparison of peak load and ductility for PUTCCS between FEM and experimental results.

Items	Experimental Value	FEM Value	(FEM Value Experimental Value)/Experimental Value
Positive peak load/kN	281.84	277.22	−1.64%
Negative peak load/kN	263.56	277.22	5.18%
Ductility	2.92	2.88	−1.37%

**Table 3:** Analysis parameters and calculation results of seam location.

Specimen	Seam Location	*P*_y_/kN	*P*_max_/kN	Δ_y_/mm	Δ_u_/mm	*μ*	Remark
SL-0	0 mm above haunch	237.35	277.22	41.62	119.90	2.88	Test conditions
SL-150	150 mm above haunch	245.25	284.46	41.49	105.04	2.53	
SL-300	300 mm above haunch	247.65	289.44	41.27	105.96	2.57	
SL-450	450 mm above haunch	249.03	289.92	42.10	106.29	2.52	

**Table 4:** Analysis parameters and calculation results of haunch height and reinforcement.

Specimen	Haunch Height/mm	Haunch Reinforcement	*P*_y_/kN	*P*_max_/kN	Δ_y_/mm	Δ_u_/mm	*μ*	Remark
HD-0-0	0	None	161.77	195.26	63.96	153.26	2.40	
HD-150-14	150	D14@200	233.72	272.10	41.38	102.98	2.49	
HD-200-12	200	D12@200	232.36	271.33	40.75	120.57	2.96	
HD-200-14	200	D14@200	237.35	277.22	41.62	119.90	2.88	Test conditions
HD-200-16	200	D16@200	241.74	281.28	42.03	115.74	2.75	

**Table 5:** Analysis parameters and calculation results of embedment depth.

Specimen	Embedment Depth/m	*P*_y_/kN	*P*_max_/kN	Δ_y_/mm	Δ_u_/mm	*μ*	Remark
DB-0	0	237.35	277.22	41.62	119.90	2.88	Test conditions
DB-2	2	243.44	284.93	40.76	113.45	2.78	
DB-5	5	254.39	295.49	41.53	106.84	2.57	
DB-10	10	263.78	298.56	42.12	103.08	2.45	

**Table 6:** Value of the input parameters.

Parameters	*U*_max_/mm	ν	*G*/MPa	*L*/mm	*B*/mm	Embedment Depth/mm	*H*/m
Value	490	0.3	100	3300	3800	3000	15

**Table 7:** Interaction coefficient *R* using Wang’s, Penzien’s, and Nishioka’s methods.

Specimen ID	SL- 0	SL- 150	SL- 300	SL- 450	HD- 0-0	HD- 150-14	HD- 200-12	HD- 200-14	HD- 200-16	DB- 0	DB- 2	DB- 5	DB- 10
Wang’s	2.68	2.68	2.68	2.68	2.72	2.68	2.69	2.68	2.68	2.68	2.68	2.67	2.67
Penzien’s	2.67	2.66	2.66	2.66	2.71	2.67	2.67	2.67	2.67	2.67	2.67	2.66	2.65
Nishioka’s	1.93	1.92	1.92	1.92	1.95	1.93	1.93	1.93	1.93	1.93	1.92	1.92	1.92

## Data Availability

The data used to support the findings of this study are available upon request from the corresponding author.
